# A novel *SLC8A1-ALK* fusion in lung adenocarcinoma confers sensitivity to alectinib: A case report

**DOI:** 10.1515/biol-2022-0090

**Published:** 2022-08-11

**Authors:** Ling Deng, Panwen Tian, Zhixin Qiu, Ke Wang, Yalun Li

**Affiliations:** Department of Respiratory and Critical Care Medicine, The First People’s Hospital Of Chongqing Liang Jiang New Area, 401121, Chongqing, China; Department of Respiratory and Critical Care Medicine, Lung Cancer Treatment Center, West China Hospital, Sichuan University, No 37 GuoXue Alley, Chengdu, 610041, Sichuan, China

**Keywords:** lung adenocarcinoma, SLC8A1-ALK, ALK fusion protein partners, alectinib

## Abstract

*ALK* fusion genes are diverse. Approximately 30 different *ALK* fusion protein partners have been described previously, and some of these fusion proteins have been reported to be effective against *ALK*-tyrosine kinase inhibitor (TKI). *ALK* rearrangements often occur at a common breakpoint in exon 20 of the genome. *SLC8A1-ALK*, a novel fusion protein partner, comes from exon 2 of the *SLC8A1* gene rearranged with exon 20 of the *ALK* gene. Here, we reported a patient with advanced lung adenocarcinoma harboring a *SLC8A1-ALK* fusion who benefited from first-line treatment with alectinib. After 2 months of taking alectinib, the targeted lung lesions and intrahepatic metastases regressed significantly. To date, the patient has achieved nearly 1 year of progression-free survival while taking the drug. Given the diversity of *ALK* fusion genes and the different efficacy of ALK-TKIs, we believe that this case report has an important clinical reference.

## 1 Introduction

The anaplastic lymphoma kinase (*ALK*) gene is located on the short arm of chromosome 2 (2p23), and it is a member of the insulin receptor superfamily that encodes the *ALK* protein. *ALK* gene rearrangement is a driver mutation underlying the development of non-small-cell lung cancer (NSCLC) that has been identified in 5–6% of cases [1]. *ALK* rearrangements are more prevalent in Asian populations of female nonsmokers [2]. Although the incidence of *ALK* rearrangement is low, 60% of patients respond well to *ALK*-tyrosine kinase inhibitors (TKIs). The most common *ALK* fusion partner in NSCLC is *EMAP*-like *4 (EML4)-ALK* (88.9%). ALK fusion with some rare genes has also been reported [3,4]. Although *ALK* fusion genes have a high response rate to *ALK*-TKIs in patients with NSCLC, some patients with rare *ALK* fusions have been reported to be non-responders to *ALK*-TKIs [5]. Zhu and He .[6] reported a case of a nonsmoking male with lung adenocarcinoma who was found to be *SLC8A1-ALK* fusion gene positive and developed resistance to treatment with crizotinib for 9 months. Given the differences in patient response to targeted *ALK* inhibitors for the treatment of lung adenocarcinoma, the genomic heterogeneity of *ALK* rearrangements has received considerable attention in recent years. So, the exploration of novel forms of *ALK* fusion and their association with tumor sensitivity to drugs remains essential. Here, we described a patient with advanced lung adenocarcinoma with a rare *ALK* gene fusion, *SLC8A1-ALK*, which was sensitive to alectinib.

## 2 Case report

**Figure 1:**
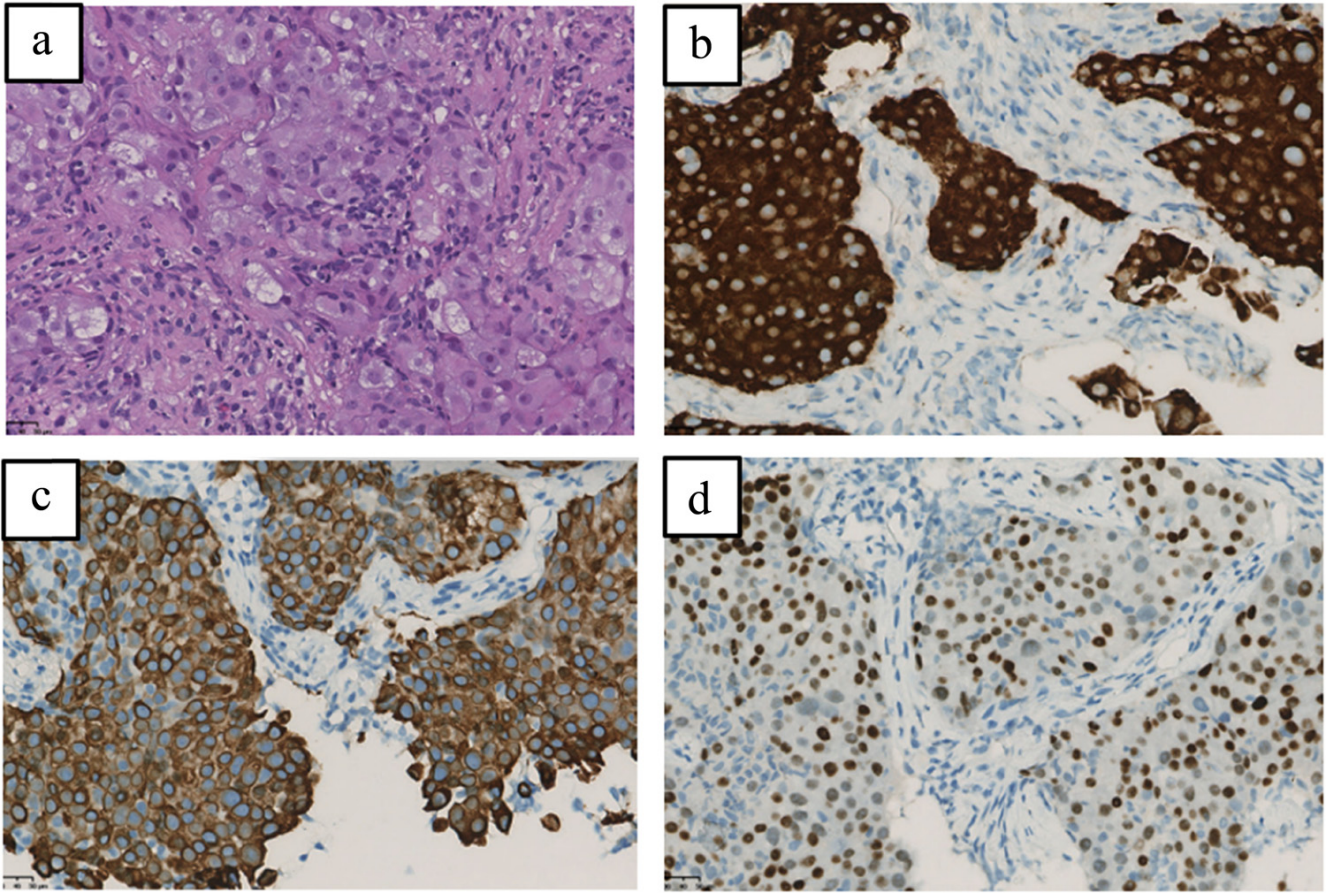
Histological findings (40× magnification). (a) Hematoxylin and eosin-stained biopsy specimen reveals adenocarcinoma; (b) immunohistochemical analysis showing ALK-positive staining; (c) keratin 7 (CK7)-positive staining; and (d) thyroid transcription factor-1 (TTF-1)-positive staining. Scale bars represent 50 μm.

**Figure 2:**
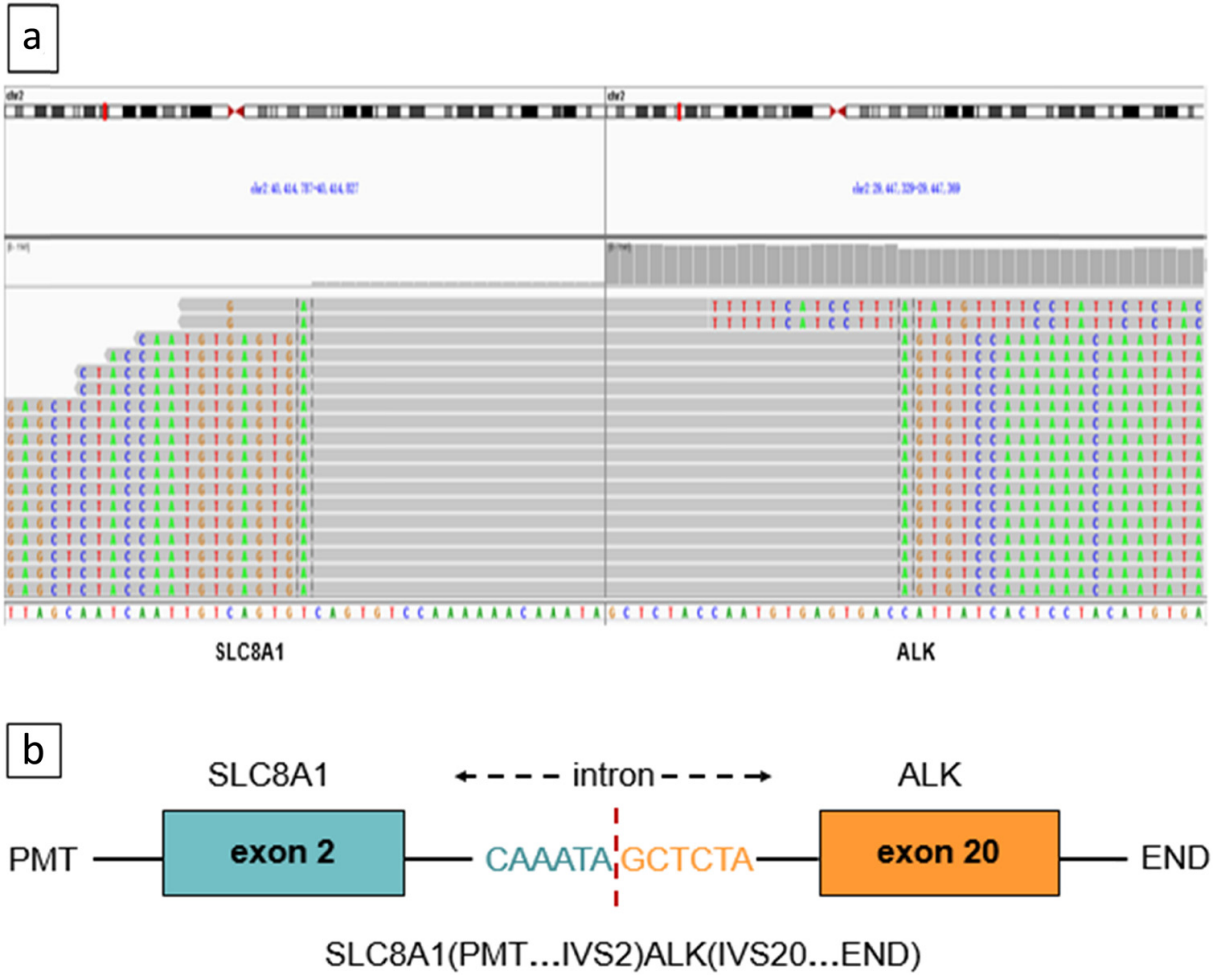
(a) Sequencing reads of *ALK* and *SLC8A1* are shown by the Integrative Genomics Viewer. *SLC8A1* and *ALK* both located on chromosome 2, the two genes translocation leading to a new fusion oncogene *SLC8A1-ALK*. (b) Schematic structure of the genomic DNA sequence shows fusion points for the *SLC8A1-ALK* fusion gene. Exon 2 of the *SLC8A1* gene rearranged with exon 20 of the *ALK* gene.

**Figure 3:**
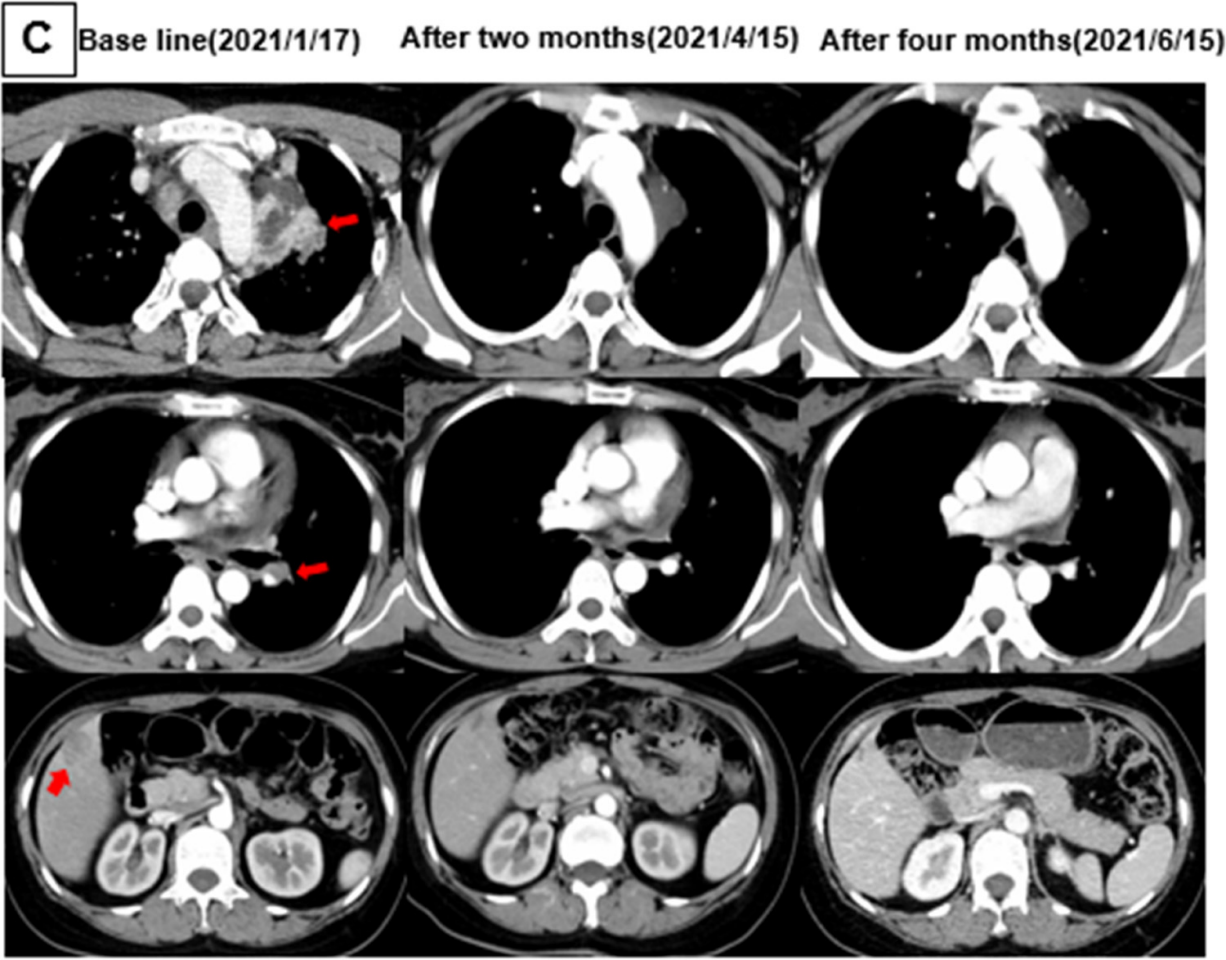
Lung adenocarcinoma shown by radiologic. Dynamic imaging of CT scan of patients treated with alectinib showing target lesions, mass invasion of the left pulmonary artery and response of liver metastases at baseline, 2 and 4 months of treatment.


A 41-year-old Chinese female nonsmoker presented to our hospital with hoarseness that had persisted for 2 months. The chest computed tomography (CT) scan showed a 5.3 cm × 5.1 cm mass in the left upper lobe with metastases to the left pulmonary artery, bilateral intrapulmonary, mediastinal, and bilateral cervical lymph nodes, liver, and multiple bones. The patient underwent fibreoptic bronchoscopy and received a pathological diagnosis of stage IVB adenocarcinoma. The immunohistochemical analysis revealed positive expression for CK7, thyroid transcription factor 1 (TTF-1), and negative expression of P63, CK(5/6), indicating an adenocarcinoma origin of the lung tumor. Besides, there was a positive staining for ALK-V and a negative staining for ROS-1 expression (Figure 1). The patient’s tumor tissues were subjected to next-generation sequencing at the DNA level based on 61 genetic panels on biopsy specimens (Nova-Seq 6000, Illumina Corporation), which revealed a rare *ALK* rearrangement, *SLC8A1-ALK* (allele frequency = 10.39%) (Figure 2a and b). Treatment with alectinib (600 mg twice daily) was initiated in February 2021. After 2 months of this therapy, CT scans were repeated, and the images showed that the lesions had obviously decreased in size to 1.9 cm × 1.7 cm (Figure 3). The tumor invasion of the left pulmonary artery and the intrahepatic metastatic lesions were also reduced. The patient was considered to have achieved partial remission (PR) according to the Response Evaluation Criteria in Solid Tumours 1.1. Patient maintains PR efficacy for 1 year after taking the drug. To date, the patient continues to take alectinib and no serious adverse effects have been observed.

**Informed consent:** Informed consent has been obtained from all individuals included in this study.
**Ethical approval:** The research related to human use has been complied with all the relevant national regulations and institutional policies and in accordance with the tenets of the Declaration of Helsinki and has been approved by the Ethics Committee of West China Hospital.

## 3 Discussion

Approximately 30 different *ALK* fusion protein partners have been described, and SLC8A1 is a rare partner for ALK. So far, Zhu and He. [6] reported a case of a nonsmoking male with lung adenocarcinoma who was found to be SLC8A1-ALK fusion gene positive by second-generation gene sequencing. This patient was selected for crizotinib as a first-line treatment option and achieved a progression-free survival (PFS) of 9 months. Following the development of resistance to crizotinib, treatment with alectinib was initiated. It can be boldly speculated that not only does the ALK fusion pattern affect the response to ALK-TKI treatment, but the same ALK fusion pattern may respond very differently to different types of ALK-TKI therapy. Of course, this requires more clinical data to discover and summarize the patterns.

Both alectinib and crizotinib were recommended as category 1 agents for first-line therapy in patients with ALK-positive NSCLC in the National Comprehensive Cancer Network (NCCN) guidelines, version 5. 2018 (7), but alectinib is preferred [7]. Alectinib is a highly selective *ALK* inhibitor that inhibits the activation of *ALK* fusion proteins and thus acts as an anti-tumor agent. The treatment with alectinib showed a survival benefit in terms of both the median PFS rate and safety compared with crizotinib in three randomized phase III studies [8]. It has also been previously reported in the literature that crizotinib is highly effective for treating typical or atypical ALK gene fusion positive for advanced NSCLC; however, few cases of observed efficacy with aletinib have been reported. We administered aletinib to the patient and so far have achieved 1 year of sustained remission. Compared with the report by Zhu and He., this suggests that PFS with first-line treatment with aletinib may be superior to crizotinib in patients with *SLC8A1-ALK* fusion lung adenocarcinoma.

The most common form of mutation in the *ALK* gene is gene translocation with another partner gene, which results in a fusion oncogene. *ALK* rearrangement produces *ALK* tyrosine kinase, which activates downstream signaling pathways and the underexpression of the *SLC8A1* gene, which leads to the dysregulation of calcium-dependent signaling pathways that promote cell proliferation and migration and inhibit cell death [8,9]. In the current case, exon 2 of the *SLC8A1* gene rearranged with exon 20 of the *ALK* gene to form a new fusion gene, *SLC8A1-ALK*. Novel *ALK* fusion proteins promote cell proliferation by activating the RAS-MAPK and JAK-STAT downstream signaling pathways through dimerization [10]. Based on the results of available studies, the *SLC8A1-ALK* fusion is considered to be tumorigenic. Our patient with this newly identified *SLC8A1-ALK* fusion benefited from alectinib treatment. We summarized the clinical characteristics of patients with NSCLC harboring rare *ALK* fusions in Table 1.

**Table 1:** The clinical characteristics of patients with NSCLC harboring rare *ALK* fusions

Article	Age/gender	Smoking status	Histologic type	ALK fusion gene	Rebiopsy specimen	Technique	*ALK*-TKIs	Response
Hao Lin, et al., 2018	56/M	Never	AC	*EML6-ALK*, *FBXO11-ALK*	Lung	NGS	Crizotinib	PR
Carlos Pagan, et al., 2018	73/M	Former	AC	*SLMAP-ALK*	Lung	NGS	Crizotinib	PR
Jing Luo, et al., 2019	44/M	Former	AC	*EML4-ALK*, *PRKCB-ALK*	Lung	NGS	Crizotinib	PR
Tingting Feng, et al., 2019	49/F	Never	AC	*TNIP2-ALK*	Lung	NGS	Crizotinib	PR
Bao-Dong Qin, et al., 2019	29/M	Former	AC	*EML4-ALK*, *BCL11A-ALK*	Bronchial	NGS	Crizotinib	PR
Chunhua Zhou, et al., 2019	43/M	Former	AC	*STRN-ALK*	Liver	NGS	Gefitinib, crizotinib	PR
Jiang-Ming Zhong, et al., 2020	60/M	Unknown	AC	*EML4-ALK*, *BIRC6-ALK*	Lung	NGS	Alectinib	PR
Hua-fei Chen, et al., 2020	52/M	Unknown	AC	*SOS1-ALK*	Lung	NGS	Crizotinib	PR
Xingyu Zhu and He, 2021	62/M	Never	AC	*SLC8A1-ALK*	Lung	NGS	Crizotinib, alectinib	PR
Hao Zeng, et al., 2021	70/M	Never	AC	*KIF5B-ALK*	Lung	NGS	Crizotinib, alectinib	PR
Present patient	41/F	Never	AC	*SLC8A1-ALK*	Lung	NGS	Alectinib	PR

AC = adenocarcinoma; F = female; M = male; *ALK-TKIs* = *ALK*-tyrosine kinase inhibitors; NGS = next-generation sequencing; PR = partial response.

## 4 Conclusion

The significance of this case report is to report not only a novel *SLC8A1-ALK* fusion in a lung adenocarcinoma patient but also the follow-up of this patient’s treatment with aletinib. It can provide a useful reference for the treatment of patients with ALK driver-positive lung adenocarcinoma.
